# The impact of methicillin-resistant *S. aureus* on length of stay, readmissions and costs: a register based case-control study of patients hospitalized in Norway

**DOI:** 10.1186/s13756-017-0232-x

**Published:** 2017-07-06

**Authors:** A. Elizabeth S. Andreassen, Caroline M. Jacobsen, BirgitteFreiesleben de Blasio, Richard White, Ivar Sønbø Kristiansen, Petter Elstrøm

**Affiliations:** 1 Norwegian Institute of Public Health, Marcus Thranesgate 2, 0473 Oslo, Norway; 2 Institute of Health and Society, University of Oslo, Norway, Forskningsveien 3A, 0373 Oslo, Norway; 3 Department of Biostatistics, Oslo Centre for Biostatistics and Epidemiology, Institute of Basic MedicalSciences, University of Oslo, Sognsvannsveien 9, 0372 Oslo, Norway; 4 Department of Antibiotic resistance and Infection control, Norwegian Institute of Public Health, Oslo, Norway

**Keywords:** Methicillin-resistant *S. aureus*, Costs, Inpatient, Diagnosis-related group, Length of stay, Readmission

## Abstract

**Background:**

Patients with methicillin-resistant *S. aureus* (MRSA) are thought to incur additional costs for hospitals due to longer stay and contact isolation. The aim of this study was to assess the costs associated with MRSA in Norwegian hospitals.

**Methods:**

Analyses were based on data fromSouth-Eastern Norway for the year 2012 as registered in the Norwegian Surveillance System for Communicable Diseases and the Norwegian Patient Registry. We used a matched case-control method to compare MRSA diagnosed inpatients with non-MRSA inpatients in terms of length of stay, readmissions within 30 days from discharge, as well as the Diagnosis-Related Group (DRG) based costs.

**Results:**

Norwegian patients with MRSA stayed on average 8 days longer in hospital than controls, corresponding to a ratio of mean duration of 2.08 (CI 95%, 1.75–2.47) times longer.A total of 14% of MRSA positive inpatients were readmitted compared to 10% among controls. However, the risk of readmission was not significantly higher for patients with MRSA. DRG based hospital costs were 0.37 (95% CI, 0.19–0.54) times higher among cases than controls, with a mean cost of EUR13,233(SD 26,899) and EUR7198(SD 18,159) respectively.

**Conclusion:**

The results of this study indicate that Norwegian patients with MRSA have longer hospital stays, and higher costs than those without MRSA.

## Background

*Staphylococcus aureus* is considered to be one of the most common pathogens causing nosocomial infections [1]. The bacterium is a normal inhabitant in the human body, found persistent on the skin or mucosa of 20% of adults [2]. It is however associated with several types of illnesses, most commonly skin and soft tissue infections, although it may also cause more severe infections [1].

In Norway, all diagnosed cases of MRSA are mandatorily notified to the Norwegian Surveillance System for Communicable Diseases (MSIS). The proportion of invasive *S. aureus* isolates in Norway that were methicillin-resistant was only 1.3% in 2012 [3], and Norway is considered to be amongst the European countries with the lowest percentage of MRSA in *S. aureus* clinical isolates.

The spread of MRSAin Norwegian hospitals is controlled through screening routines, which consist of testing patients before admission if they have previously tested positive or are suspected of having been exposed to MRSA in the 12 months prior. The current MRSA-strategy in Norwegian hospitals consists of isolating suspected and confirmed MRSA positive patients, work restrictions for healthcare personnel who test positive for MRSA, and decolonization of carriers [4]. Some studies have explored the cost-effectiveness of drugs used to treat MRSA infections and costs related to MRSA screening in Norway [5, 6].To our best knowledge, there are currently no studies estimating the economic impact of MRSA positive patients based on LOS, readmissions or DRG costs in Norwegian hospitals.

The aim of our study was to estimate the resource use associated with MRSA in Norwegian hospitals in terms of length of stay, readmissions within 30 days and DRG-based costs, in order to provide a better knowledge base for infection prevention strategies.

## Methods

This was a register-based matched case-control study with patients admitted to hospital during the year 2012.

We used data from the South-Eastern Norway health region for the period January 1 to December 31, 2012, provided by the Norwegian Patient Registry (NPR) and the Norwegian Surveillance System for Communicable Diseases (MSIS). This region represents approximately 56% of the Norwegian population, including the capital city of Oslo, and comprises 18 hospitals. Using the unique national identification number assigned to each Norwegian citizen, information from both the NPR and MSIS databases could be linked in order to extract the MRSA status of inpatients that had been tested for MRSA. Patients who have undergone screening because they met at least one of the screening criteria, but had a negative MRSA-test, are not registered in MSIS. Ethical approval was obtained from the South-Eastern Regional Committee for Medical and Health Research Ethics (REC): 2013/1004/REK.

**Fig. 1:**
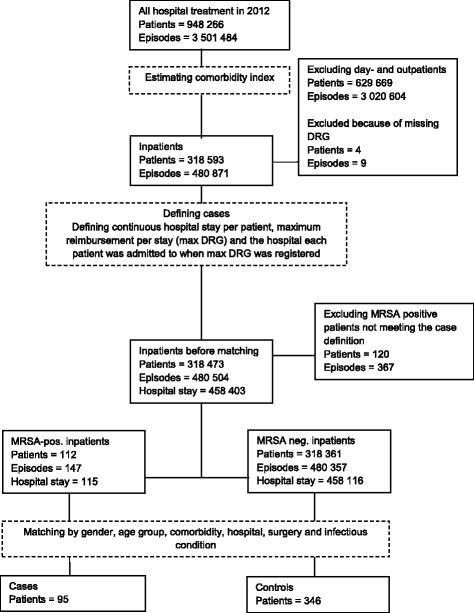
Sample selection flowchart. Cases and controls were selected from a merged dataset including all patients who had received health care services in hospitals of the South-Eastern Norway health region (provided by the Norwegian Patient Registry) and all notified cases of methicillin-resistant *S. aureus* (provided by the Norwegian Surveillance System for Communicable Diseases) during the year 2012. Only patients who were diagnosed MRSA-positive at the earliest eight days prior or during their hospital stay, were included as cases. In total, 82 (86%) cases were matched to four control patients; the rest had less than four controls per case

The dataset included individual patients, at all levels of care (inpatient, outpatient, day patient), aggregated by episode of care. We identified inpatient stays and defined those hospital stays by aggregating all episodes of care with continuous dates (Fig. 1). Only the first hospital stay recorded for 2012 was considered in the analysis. MRSA-cases were defined as patients diagnosed with MRSA at the earliest eight days prior to hospitalization, or in the course of their hospital stay. All patients diagnosed with MRSA in Norway are offered treatment before admission to hospital. This treatment together with microbiological analyses, takes a minimum of nine days. By restricting the case definition to a positive sample within this time frame, we maximized certainty that cases were MRSA positive during the hospital stay. Patients missing DRG codes, MRSA positive patients diagnosed more than eight days prior to hospitalization, and those patients without an exact match were excluded from the analysis. The flow of patient inclusion and exclusion is presented in Fig. 1.Controls were selected from the group of patients not diagnosed with MRSA.

Cases were randomly matched, one to four controls, based on 10-year age groups, gender, hospital, surgery, whether they were diagnosed with an infection or not during the stay and Charlson Comorbidity Index (CCI) [7] (Table 1). For 13 cases (14%) it was not possible to obtain four controls; among these, one case was matched with three controls, three with two controls and nine with one control patient. Among those patients who were transferred between hospitals, the matching was done based on the hospital where the highest DRG cost weight was registered. We used the DRG codes indicating surgical operations to identify patients who underwent surgery. In addition, we performed analyses of subgroups where we combined ICD-10 codes (registered during a hospital stay) with data from MSIS. The MSIS data include MRSA diagnosis (colonization or infection), sample material and clinical picture at the time of notification (Table 2).

**Table 1:** Description of included patients

	Cases (*n* = 95)	Controls (*n* = 346)
Variables	*n (%)*	*n (%)*
Male gender	49 (52%)	179 (52%)
Age		
0–9	16 (17%)	64 (18%)
10–19	3 (3%)	9 (3%)
20–29	5 (5%)	20 (6%)
30–39	8 (8%)	29 (8%)
40–49	9 (9%)	28 (8%)
50–59	14 (15%)	44 (13%)
60–69	14 (15%)	56 (16%)
70–79	14 (15%)	51 (15%)
80–89	11 (12%)	41 (12%)
> 89	1 (1%)	4 (1%)
Comorbidity index		
	68 (72%)	251 (73%)
1	11 (12%)	39 (11%)
2	13 (14%)	44 (13%)
3	2 (2%)	8 (2%)
8	1 (1%)	4 (1%)
Hospitals (anonymized)		
A	20 (21%)	74 (21%)
B	18 (19%)	70 (20%)
C	8 (8%)	32 (9%)
D	8 (8%)	26 (8%)
E	7 (7%)	28 (8%)
F	6 (6%)	23 (7%)
G	6 (6%)	22 (6%)
H	4 (4%)	16 (5%)
I	4 (4%)	13 (4%)
J	4 (4%)	13 (4%)
K	3 (3%)	12 (3%)
L	2 (2%)	8 (2%)
M	2 (2%)	2 (1%)
N	1 (1%)	4 (1%)
O	1 (1%)	2 (1%)
P	1 (1%)	1 (0%)
Infections		
No infections	42 (44%)	185 (53%)
Infection	53 (56%)	161 (47%)
Non-severe infections	31 (33%)	100 (29%)
Severe infections	15 (16%)	50 (14%)
Procedure		
Surgery	17 (18%)	48 (14%)

**Table 2:** Infections based on ICD-10 codes recorded for cases and controls during the first hospital stay

Infections	Infection types (ICD-10 codes)
All	Intestinal infectious diseases (A04; A08); Tuberculosis (A15); Sepsis (A41); Erysipelas (A46); Bacterial infection of unspecified site (A49); Human immunodeficiency virus disease (B23); Cytomegaloviral disease (B25); Acute and subacute endocarditis (I33); Acute upper respiratory infections (J03; J06); Pneumonia/ other acute lower respiratory infections (J15; J18; J20; J21); Abscess of anal and rectal regions/ peritonitis (K61; K65); Infections of the skin and subcutaneous tissue (L02 – L04; L08); Infectious arthropathies (M02); Necrotizing fasciitis (M726); Osteomyelitis (M86); Acute tubulo-interstitial nephritis (N10); Urinary tract infection, site not specified (N390); Inflammatory disorders of breast/ infections of breast associated with childbirth (N61; O91) Infection following a procedure, not elsewhere classified or due to internal fixation device (T814; T846)
Infections possibly caused by *S. aureus*	
Non-severe infections	Bacterial infection of unspecified site (A49); Acute tonsillitis, unspecified (J039); Acute upper respiratory infections of multiple and unspecified sites (J06); Abscess of anal and rectal regions (K61) Cutaneous abscess, furuncle and carbuncle (L02); Cellulitis (L03); Other local infections of skin and subcutaneous tissue, unspecified (L08); Urinary tract infection, site not specified (N390); Inflammatory disorders of breast/ Infections of breast associated with childbirth (N61; O91)
Severe infections	Other sepsis (A41); Acute and subacute endocarditis (I33); Bacterial pneumonia, unspecified (J159); Pneumonia, organism unspecified (J18); Necrotizing fasciitis (M726); Osteomyelitis (M86); Acute tubulo-interstitial nephritis (N10); Infection following a procedure, not elsewhere classified or due to internal fixation device (T814; T846)

The comorbidity index was estimated for each patient according to the method developed for economic evaluations by Charlson et al., based on diagnoses as described by Quan et al. [8, 9]. The Canadian version of the International Coding of Disease 10th revision (ICD-10) used by Quan et al., was validated against the Norwegian ICD-10 codes used in this study. Due to data availability, we used all the ICD-10 codes registered in 2012 to define CCI instead of using previous years’ diagnoses. This included diagnoses registered at both inpatient and outpatient episodes of care.

The outcome variables were length of stay (LOS) of the first hospitalization, readmissions within 30 days from discharge, and costs based on the highest Diagnosis-Related Group (DRG) cost weight during the first hospital stay. Estimation of readmissions within 30 days was restricted to patients discharged before December 1, 2012.

**Fig. 2:**
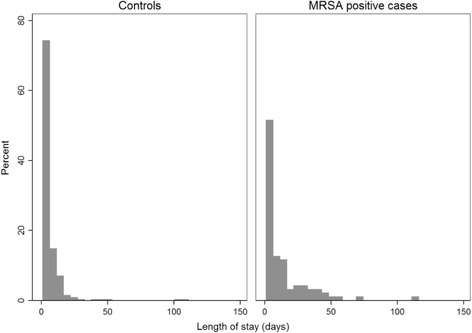
Distribution of LOS for cases and controls based on the first hospital stay in 2012. The number of days spent in hospital for the first hospital stay registered in 2012 showed that patients not diagnosed with methicillin-resistant *S. aureus* were more likely to have shorter hospital stays than patients diagnosed with methicillin-resistant *S. aureus*. *LOS,* length of stay

**Fig. 3:**
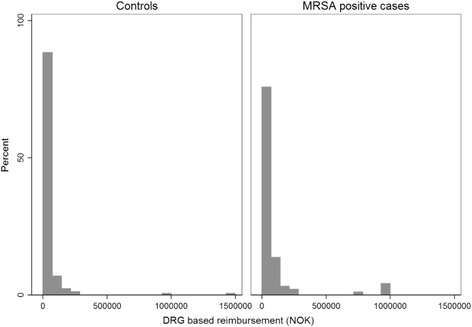
Distribution of DRG based costs for cases and controls during their first hospitalization in 2012. DRG cost weights are the basis for a considerable share (40%) of hospital reimbursement in Norway, and hence, an important proxy for costs per patient, however inaccurate. Due to limited access to micro costing data for patients in this study, DRG cost weights were used to estimate costs per hospital stay, although these costs are potentially an underestimate of the actual economic burden caused by MRSA to hospitals

All statistical analyses were performed in Stata Statistical Software (StataCorp, 2013). The distribution of patients in each group were presented as proportions. We estimated median in addition to mean for the outcome variables since the data were not normally distributed. Given a matching ratio of 1:4 and thus data on both group and individual level, we measured the outcomes using Multi-level Mixed effect Regression models. Having skewed distributions (Figs. 2 and 3), we analyzed LOS using negative binomial regression, readmissions with both logistic and linear regression, and costs with log of the linear regression outcome [10].

## Results

The dataset included a total of 948,266 patients. Of those, 318,593 were inpatients (Fig. 1). Before matching, 4 patients missing DRG codes and 120 MRSA positive patients who were diagnosed with MRSA more than eight days prior to hospitalization were excluded. Additionally, 17 patients who met the inclusion criteria did not have an exact match with other patients and were excluded from the analysis.In total, 95 MRSA positive patients met the inclusion criteria for cases, and were matched with 346 MRSA negative controls. Gender was equally represented in both groups (males 52%).Overall, 75% of the study population consisted of children less than 10 years and adults over 50 years of age. The majority (84%) had a CCI of <2. In total, 65 (15%) of the patients underwent surgery while admitted (Table 1). Based on the type of hospital, 44 (10%), 301 (68%) and 98 (22%) patients were hospitalized in teaching hospitals, general hospitals with more than 250 beds, and local hospitals with less than 250 beds, respectively.

The median LOS was 6 (IQR 3.00–15.00) days for MRSA positive patients and 4 (IQR 2.00–7.00) days for MRSA negative patients, while the means were 14 (SD 17.24) and 6 (SD 9.61) days, respectively.The greatest difference in LOS between cases and controls was observed in the subgroup with no infections and the group diagnosed with severe *S. aureus* infections (Table 3). The distribution of LOS is shown in Fig. 2.

**Table 3:** Univariate analysis of LOS distributed by infectious condition

Length of stay	Cases (n = 95)		Controls (n = 346)		Negative binomial regression		
	Median (IQR)	Mean (SD)	Median (IQR)	Mean (SD)	Ratio of mean duration	95% CI	*p*-value
All patients (*n* = 95/441)	6.00 (3.00–15.00)	13.71 (17.24)	4.00 (2.00–7.00)	6.05 (9.61)	2.08	1.75–2.47	<0.05
• No infections (*n* = 42/227)	9.50 (3.00–23.00)	14.36 (13.87)	3.00 (2.00–6.00)	5.75 (9.03)	2.48	1.91–3.21	<0.05
• Any infection (*n* = 53/214)	5.00 (3.00–13.00)	13.19 (19.62)	4.00 (2.00–7.00)	6.40 (10.25)	1.58	1.26–1.98	<0.05
- Non-severe infections (*n* = 31/131)	5.00 (3.00–9.00)	8.94 (19.27)	3.00 (2.00–5.00)	5.18 (10.84)	1.40	1.02–1.92	<0.05
- Severe infections (*n* = 15/65)	14.00 (5.00–36.00)	21.60 (20.69)	5.00 (4.00–12.00)	8.88 (9.70)	1.86	1.33–2.62	<0.05

*IQR* Interquartile range, *SD* Standard deviation, *CI* Confidence interval

A higher proportion of cases were readmitted within 30 days compared to controls, but overall there was no statistically significant difference regarding readmissions (Table 4).

**Table 4:** Univariate analysis of readmissions within 30 days distributed by infectious condition

Readmissions within 30 days	Cases (*n* = 74)	Controls (*n* = 299)	Logistic regression			Linear regression		
	n (%)	n (%)	OR	95% CI	*p*-value	Coef.	95% CI	*p*-value
All patients (*n* = 74/299)	10 (14%)	22 (10%)	1.50	0.65–3.46	0.34	0.04	−0.04 – 0.12	0.34
• No infections (*n* = 30/144)	4 (13%)	15 (13%)	1.02	0.31–3.32	0.98	0.00	−0.13 – 0.14	0.98
• Any infection (*n* = 44/155)	6 (14%)	7 (6%)	3.44	0.79–15.00	0.10	0.08	−0.01 – 0.17	0.07
- Non-severe infections (*n* = 25/83)	4 (16%)	1 (2%)	10.86	1.15–102.77	<0.05	0.14	0.04–0.25	<0.05
- Severe infections (*n* = 13/57)	2 (15%)	5 (11%)	1.76	0.19–16.67	0.62	0.04	−0.13 – 0.21	0.62

*OR* odds ratio, *CI* confidence interval

In total, cases had higher DRG-based costs than controls, with a median of EUR5174(IQR 3347–9059) and EUR3762 (IQR 2405–6669), respectively. In the subgroup of patients with infections, the median costs were almost equal, although among patients with severe infections the mean costs were significantly higher for cases than controls (Table 5). The distribution of DRG based costs is shown in Fig. 3.

**Table 5:** Univariate analysis of DRG based costs distributed by infectious condition

Reimbursement (EUR)	Cases (*n* = 95)		Controls (*n* = 346)		Linear regression with log(reimbursement) as outcome		
	Median (IQR)	Mean (SD)	Median (IQR)	Mean (SD)	Coef	95% CI	*p*-value
All patients (*n* = 95/441)	5174 (3347–9059)	13,233 (26,899)	3762 (2405–6669)	7198 (18,159)	0.37	0.19–0.54	<0.05
• No infections (*n* = 42/227)	6423 (3603–8445)	13,208 (26,850)	3680 (2103–6669)	7797 (22,619)	0.48	0.22–0.74	<0.05
• Any infection (*n* = 53/214)	4560 (3148–11,178)	13,252 (27,195)	4355 (3148–6326)	6511 (11,033)	0.21	−0.03 – 0.45	0.09
- Non-severe infections (*n* = 31/131)	3450 (2523–5794)	8065 (18,243)	3347 (2523–5794)	5299 (12,802)	0.09	−0.25 – 0.44	0.60
- Severe infections (*n* = 15/65)	7600 (5794–21,931)	26,904 (41,802)	7600 (4560–7600)	9300 (7268)	0.47	0.15–0.79	<0.05

*IQR* Interquartile range, *SD* Standard deviation, *CI* Confidence interval, *EUR* Euro, Exchange rate NOK-EUR7.4805 average for 2012

## Discussion

The results of this study indicate that Norwegian patients with MRSA had longer hospital stays, and higher costs than those without MRSA. We did not find statistically significant differences concerning readmissions.Our study is the first in Norway addressing the economic burden of MRSA in hospitals, and one of few studies that estimate the costs of MRSA in general, not only the life threatening MRSA related conditions.

We found that MRSA positive patients stay longer in the hospital than their controls, and this is statistically significant both for the total study population and subgroups. Previous studies have shown that patients with severe MRSA infections have longer hospital stays compared to patients with severe MSSA infections [11–14]. A result of particular interest in this study was that we found the largest difference in LOS among patients without infections. In Norway, this result may be a consequence of the comprehensive infection control measures applied in hospitals, such as single room isolation of patients with MRSA. Some studies have addressed the possible negative consequences of single room isolation in hospitals; for example isolation may contribute to delayed examination and treatment of patients, as well as poorer follow up (less supervision, worsening of condition due to delayed treatment) [15].

Readmission within 30 days from discharge is used as an indicator of quality of hospital care in Norway. We observed few readmissions overall, and even fewer in patients with severe infections. The results showed only small and not statistically significant differences in risk of readmission among cases and controls. Interestingly, although our results indicated that isolation adds to prolonged hospital stay, we did not find that isolation leads to poorer quality of medical treatment as measured by the risk of readmission. The results must be interpreted cautiously as the sample is quite small.

Median and mean costs were higher for cases than controls overall. However, the median costs in the subgroups of patients with infections, were approximately equal. Claudia Hubner et al. used an average daily reimbursement per bed based on the actually claimed G-DRG in the patient record, to estimate opportunity cost of blocked beds. They also accounted for empirical costs related to hygienic measures and laboratory use, and found MRSA-attributable costs of EUR 8673 per case [16].The DRG system is not designed to estimate real costs of each individual patient, but specific information regarding the patient’s age, gender, diagnoses and treatments, is used to categorize patients into cost groups. We aimed to get cases and controls as similar as possible, where MRSA was the only distinguishing factor. Most cases and controls have, consequently ended up in the same cost groups. None of the cases in this study were registered with DRG codes specifically reflecting the finding of resistant bacteria.

Taking into consideration that the DRG system does not account for MRSA status and the additional costs of infection control measures, such as screening and isolation of MRSA patients, the results in this study are most likely an underestimate of the actual cost differences.The Norwegian infection control measures are comprehensive and costly, and they are applied to both colonized and infected patients. Jinshuo Li estimated that the cost of resources associated with isolation were approximately 2012 EUR 856 per day [5].In addition, our results showed that MRSA positive patients had longer hospital stays than patients without MRSA. The average cost of a patient per day in Norwegian hospitals in 2012 was estimated to be EUR 5368 [17].

The strengths of this study were the quality and accuracy of the dataset utilized for analyses. The dataset included the actual LOS for each episode of hospitalization for all patients in the health region. Norwegian health registries provide information on all the patients, their unique encrypted identification number allows tracking them as well as linking data from different health registries. Therefore, despite a small number of cases, it gives a complete overview of the diagnosed MRSA cases in the health region. The Norwegian surveillance system contains all patients who have been diagnosed with MRSA, regardless of where the test is taken. However, most patients in Norwegian hospitals are not routinely screenedfor MRSA, which may lead to an underestimation of the true MRSA rate and undetected MRSA colonization in controls. The distributions of LOS and costs (Figs. 2 and 3) show distributions heavily skewed to the right, with a mean close to zero. Based on the distribution of the outcome variable in the study population, it is likely that MRSA positive patients among controls have ended up close to the mean value, and hence would not have significantly influenced the results. However, if any MRSA positive controls are outliers, they have consequently masked a larger difference between cases and controls.

Two other European studies have used DRG cost weights in addition to other costs [14, 16]. Healthcare resource utilization measurements that are not included in the hospital reimbursement DRG, were not available through the registry data used in our study. For this reason, we do not know the total cost and resource use of this population. Considering that MRSA-positive patients are subject to very strict infection control routines in Norway and have extended LOS compared with controls, the cost difference between cases and controls is potentially greater.Norwegian health registries are intended for administrative purposes and are not designed for research, therefore, although we used registry data of high quality in our study, we were limited in our scope of analysis by the measurements available. The study design implies that there are uncontrolled risk factors. We attempted to control for potential confounders thought to be relevant by matching.

## Conclusion

This study shows that MRSA contributes to longer length of stay in hospitals and higher costs based on DRG cost weights. It gives an improved knowledge base with regards to the consequences of resistant bacteria, and may help Norwegian policy makers to make informed decisions concerning resource allocation, infection prevention programs and guideline development. However, in order to get a better understanding of the costs related to resistant bacteria, micro-cost studies are needed.

## Abbreviations

CCI: Charlson comorbidity index
DRG: Diagnosis-related group
ICD-10: International coding of disease 10th revision
LOS: Length of stay
MRSA: Methicillin-resistant *Staphylococcus aureus*
MSIS: Norwegian surveillance system for communicable diseases
NPR: Norwegian patient registry
REC: South-Eastern regional committee for medical and health research ethics
